# Epidural blood patch for refractory low CSF pressure headache: a pilot study

**DOI:** 10.1007/s10194-011-0331-7

**Published:** 2011-04-02

**Authors:** Søren Aalbæk Madsen, Jonna Storm Fomsgaard, Rigmor Jensen

**Affiliations:** 1 Intensive Care Unit 4131, National Hospital, University of Copenhagen, Blegdamsvej 9, 2100 Copenhagen, Denmark; 2 Department of Anesthesiology, Glostrup Hospital, University of Copenhagen, Ndr. Ringvej 57, 2600 Glostrup, Denmark; 3 Danish Headache Center, Department of Neurology, Glostrup Hospital, University of Copenhagen, Ndr. Ringvej 57, 2600 Glostrup, Denmark

**Keywords:** Intracranial hypotension, Epidural blood patch, Headache, Low CSF pressure headache

## Abstract

Once believed an exceedingly rare disorder, recent evidence suggests that low cerebrospinal fluid (CSF) pressure headache has to be considered an important cause of new daily persistent headaches, particularly among young and middle-aged individuals. Treatment of low CSF pressure headache consists of non-invasive/conservative measures and invasive measures with epidural blood patch providing the cornerstone of the invasive measures. In the present pilot study we therefore aimed to evaluate the treatment efficacy of epidural blood patch (EBP) in treatment-refractory low-pressure headache. Our primary effect parameter was total headache burden defined as area under the curve (AUC: intensity × duration) and as secondary effect parameters we identified: intensity (VAS 0-10), frequency (days per month), duration in hours (total hours/month) and also medication days (days on medication/month). In our primary effect parameter we found a significant reduction in AUC with more than 25% and this is considered to be clinically relevant. We found also a significant and relevant reduction at −22% in intensity. A trend towards reduction in duration was seen. We found no statistically significant reduction in frequency. An increase in days with use of medication was found. Increased awareness of low CSF pressure headache is emphasized and a controlled larger randomized study is needed to confirm the results. However the present results, allows us to conclude that EBP in treatment-refractory low CSF pressure headache can be considered as a treatment option.

## Purpose

To evaluate the treatment efficacy of epidural blood patch (EBP) in treatment-refractory low CSF (cerebrospinal fluid) pressure headache.

## Introduction

Although first described in 1938 low CSF pressure headache is still an under-diagnosed headache disorder. This being partly due to unfamiliarity, and with the overlap with other forms of headache, often leading to misdiagnosis, incorrect treatment and leaving the patient with a persistent severe headache [1]. Low CSF pressure headache has significant symptom-overlap with migraine, tension type headache, post-infectious headache and most commonly post-dural puncture headache [2]. Symptoms vary greatly but the orthostatic headache, tinnitus, hypacusis, photophobia and/or nausea indicating low CSF pressure are the most frequently reported [4]. However, the associated symptoms may vary ranging from classic neurological symptoms to cognitive defects [3]. Diffuse pachymeningeal enhancement on brain magnetic resonance imaging (MRI) is the major finding of the classic syndrome. However, the diagnosis maybe difficult in long lasting cases as the cardinal symptom, the orthostatic headache-feature, may disappear over time and the diffuse meningeal enhancement may be absent. Over time various terminologies has been used to describe the syndrome:

- Spontaneous (or idiopathic) low CSF pressure headache
- Spontaneous (or primary) intracranial hypotension
- Low CSF volume headache
- Hypoliquorrhoeic headache
- Aliquorrhea
- CSF leak headache
- CSF hypovolemia
- CSF volume depletion.

The accepted etiology is leakage of cerebrospinal fluid, either spontaneously or as a result of minor/trivial trauma, instrumentation of or near the structures surrounding the spinal cord. The leak is usually located in the upper thoracic level or at the cervico-thoracic junction [3]. As a result of this leakage, the brain sags providing traction on bridging veins, pain-sensitive meningeal structures and the cranial nerves, possibly explaining the associated auditory and, at times visual symptoms. In accordance with the Monroe–Kellie doctrine, secondary venodilation may develop as a compensatory measure to the low CSF pressure, adding a vascular component with venous dilatation to the typical MR-presentation introduced as SEEPS (subdural hygroma, enhancement, engorged veins, pituitary hyperaemia, and sagging of the brain [3]). Once believed to be an exceedingly rare disorder, recent evidence suggests that low CSF pressure headache is not that rare and has to be considered an important cause of new daily persistent headaches, particularly among young and middle-aged individuals [4]. This said few data are available regarding the demography of low CSF pressure headache. A peak incidence centered around 40 years of age has been reported, the incidence is estimated to 5 per 100,000 and women are overrepresented in a 2:1 ratio [3].

Treatment of low CSF pressure headache consists of non-invasive/conservative strategies and/or invasive measures with epidural blood patch providing the cornerstone of the invasive measures. Although no randomized clinical trials are available to evaluate the effectiveness of the various treatment protocols, initial treatment for most patients consist of conservative measures. The most conservative measure, bed rest and time, is probably effective in many patients [3]. Analgesics are often employed early as part of the conservative treatment they do though provide little relief. Steroid-therapy is of anecdotal value [4].

Apart from EBP, other invasive measures are surgical repair and epidural fibrin glue, they are though, limited by the fact that the epidural lesion has to be located which is not always possible [3]. EBP is the mainstay in the treatment of low CSF pressure headache, but no protocol has yet been developed. Treatment protocols vary between injection of 10–100 ml autologous blood and 1–6 EBPs, placement in the Trendelenburg position, premedication, etc. [5]. In the present pilot study we therefore aimed to evaluate the treatment efficacy of epidural blood patch (EBP) in treatment-refractory low-pressure headache.

## Methods

We followed 14 patients all diagnosed at the Danish Headache Center (DHC), the national tertiary headache center, with low CSF pressure headache and treated with EBP. All the patients followed in this pilot study presented themselves with an orthostatic component as part of their symptoms although an overlap in symptoms was seen. At the time of initial evaluation, and at the time of the EBP all patients, except two, described significant worsening of the headache either when erect or when physically active.

All patients had been refractory to conservative non-invasive management and were referred to DHC for further evaluation. Before their first visit all patients receive a diagnostic headache diary and are told to record headache characteristics, including type and amount of medication used, prospectively for at least a 4-week period. They also fill out a questionnaire regarding status of health, impact on work, family and social life and prior headache treatment.

A detailed headache history is obtained by means of a standardized procedure at the initial consultation. The history is supplemented by the diagnostic headache diary and the general medical questionnaire. After a complete general physical and neurological examination, all first-visit patients were classified according to ICHD-II by a headache specialist.

The headache diaries of the 14 patients were reviewed prior to treatment with EBP and a second review at least 12 months after treatment with EBP. Additional phone-interviews were performed and data from the preliminary and final examination were extracted in case of missing data. Our primary effect parameter was total headache burden defined as area under the curve (AUC: intensity × duration) and as secondary effect parameters we identified: intensity (VAS 0-10), frequency (days per month), duration in hours (total hours/month) and also medication days (days on medication/month).

All EBP, but two, were performed by the same senior anesthesiologist (J.F.) according to a standardized procedure after obtaining full informed consent. All EBPs were performed under strict aseptic conditions in an operating room. The EBPs were all placed at the lumbar level (L2/3, L3/4, and L4/5). All EBP were performed with 18-G Touhy needle, using a midline approach with the patients lying on one side. Autologous blood (6–37 ml; mean 23.64 ml) was injected until patients complained of lumbar pain. After the procedure the patients were placed supine in bed for the next 24 h.

## Results

In the period of 2005–2010 we intended to treat 25 patients with EBP on the indication of treatment-refractory low CSF pressure headache. During the follow-up process seven patients were excluded due to incomplete pre-treatment data, one patient did not fill out post-treatment headache diary due to ongoing serious illness, one patient withdrew consent and two patients failed to return post-treatment headache diaries.

After the follow-up period our study consisted of 14 patients; 2 women and 12 men, aged 33–71 years of age (mean 52.86). All were treated and observed in the period 2005–2010. All received EBP 177–6,570 days (mean 1,779.29 days) after debut of headache. Follow-up period was 415–1,759 days (mean 1,258.4 days). Further patient characteristics are displayed in Table 1.

**Table 1:** Patient characteristics (*N* = 14)

Patient	Gender	Age (years)	Eliciting cause/EBP indication	ml blood injected (1st/2nd)	Time in days from debut to EBP	Follow-up period in days after last EBP
M1	Male	62	SIH	16	300	1,639
M2	Male	57	SIH	25	180	1,350
F1	Female	63	Iatrogenic (lbp)	20	387	1,890
M3	Male	59	Iatrogenic (post op.)	25/25	3,600	1,677
M4	Male	47	SIH (sneeze)	25/25	1,215	1,759
M5	Male	35	SIH	35	5,110	529
M6	Male	37	Iatrogenic (lbp)	23	438	798
M7	Male	56	Posttraumatic (moderate)	23	6,570	1,738
M8	Male	62	SIH	30/30	730	615
M9	Male	57	SIH	15	1,278	1,336
M10	Male	56	SIH	25/25	180	1,209
M11	Male	71	SIH	17/25	4,015	1,746
M12	Male	45	Posttraumatic (moderate)	30	730	976
F2	Female	33	SIH (labor)	22/12	177	415
Total (*N* = 14)	12 M/2F	52.9 years			1,779.3	1,258.4

Despite a number of eliciting causes all patients had significant overlap in symptoms and orthostatic headache as their core symptom. Nine patients presented with symptoms of spontaneous intracranial hypotension (SIH), hereof two patients with a history of tension type headache and medication overuse headache, and another two patients reported Valsalva maneuver (sneeze, labor) as eliciting cause. Two patients reported physical trauma as the eliciting cause. The traumas would be categorized as moderate. One patient was struck by a falling bookcase, resulting in laceration to the forehead and unconsciousness. The other trauma was caused by a falling loading ramp on a lorry also resulting in laceration to the forehead and unconsciousness. Three patients reported the eliciting cause as iatrogenic (lumbar puncture or post-operative).

All patients reported a significant orthostatic component as an initial part of their headache. Other symptoms included fatigue, tinnitus, photophobia, phonophobia, neck stiffness and concentration problems.

**Fig. 1:**
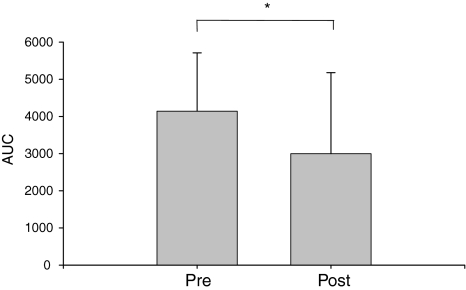
Box plot AUC pre-EBP versus post-EBP

As our primary effect parameter total headache burden was defined as AUC (mean daily intensity × summated daily duration) and a statistically significant reduction was identified in AUC [pre-AUC 4,139.1 (±419.8 SEM) vs. post-EBP AUC 2,997.2 (±582.7 SEM); paired *t* test *p* = 0.015 (CI 265.7; 2,018.1)] (see Fig. 1).

**Fig. 2:**
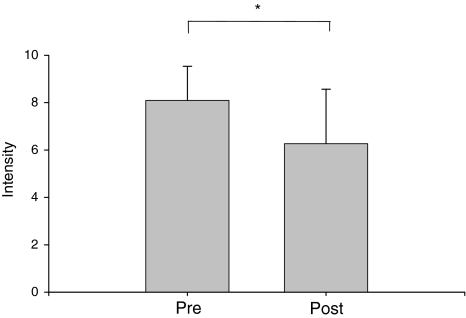
Box plot. Intensity pre-EBP versus post-EBP

As secondary effect parameters we identified intensity (mean daily VAS score) and found a statistically significant reduction [pre-EBP intensity 8.1(±0.4 SEM) vs. post-EBP intensity 6.2 (±0.6 SEM)]. This represents a reduction of 22% in intensity on VAS 0-10 scale (see Fig. 2).

**Fig. 3:**
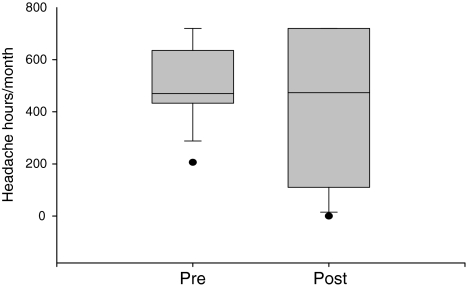
Box plot. Duration in headache hours/month

We found no statistically significant reduction in frequency, defined as days with headache (Wilcoxon signed rank test, *p* = 0.313). Furthermore, a trend, but not statistically significant, a reduction in duration (defined as total hours of headache per month) was noted [pre-EBP duration 507.9 (±39.4 SEM) vs. post-EBP 421.6 (±71.7 SEM); paired *t* test: *p* = 0.11 (CI −194.9; 22.2)] (see Fig. 3).

An increase in days with use of medication [pre-EBP days 10.0 (Q 0;24) vs. post-EBP days 26.5 days (Q 1;30); Wilcoxon signed rank test: *p* = 0.049] was found.

## Discussion

We intended to evaluate the treatment efficacy of epidural blood patch in long-lasting treatment-refractory low CSF pressure headache. In total, 25 patients were reviewed but due to losses in the follow-up period we ended up including 14 patients.

In our primary effect parameter, AUC, we found a significant reduction with more than 25% and this is considered to be clinically relevant. Likewise a significant and relevant reduction in intensity at −22% was identified.

As expected, although statistically not significant, a trend towards reduction in duration was seen, whereas the headache frequency was unchanged. It can be theorized that lack of power is the reason for behind this. The increase in days with use of medication was expected due to a low number of patients with medication overuse and to the initiation of optimized treatment strategies in a subset of patients.

The treatment of low CSF pressure headache has never been evaluated by randomized clinical trials [3], but several series have now documented positive results for EBP in treating SIH [6–10]. These series report a success rate of 36–90% of complete recovery after one EBP increasing with the numbers of EBPs. Our series does not quite support these results. Our patient population differed from other series in very long headache history, male preponderance and higher age. Although noticeable, neither age-specific nor gender-specific treatment response has ever been documented. Injected volume was very similar among studies.

We treated all our patients with lumbar EBP and placed them in a supine position for 24 h to ensure sufficient spread. Ferrante [5], Horikushi [6] and Berroir [7], etc. [8, 9] primarily performed lumbar EBPs but used different post-EBP treatment regimens. Thus, neither age, gender, site-specific EBP, nor post-EBP regimen seems to explain the differences found between this series and earlier published results.

A marked difference in this series compared to other publications is the timing of EBP treatment. In this study a mean of 1,779.29 days (177–6,570) passed from symptom onset to EBP treatment in our center*.* Other series report a mean of 20–62 days [5, 7–9], Chung et al. [10] performed EBP treatment after 5–7 days of supportive treatment but time from symptom onset is unclear. This delay in treatment in our patients was due to further diagnostic workup and limitations in acute admissions availability. Also these long lasting patients may be more subtle and differently affected than the acutely and severely affected patients. Therefore, increased awareness and a detailed history of onset of headache is of major importance for the diagnosis, and some low CSF pressure headache patients may in fact be overlooked because of their atypical presentation. Senchakova et al. suggested that the EBP procedure was more effective the more rapidly it was performed after symptom onset, and that persisting symptoms resulted in slower and incomplete recovery. This suggests a chronification of pain or dysregulation of the intracranial blood volume [8]. Additionally, it is well known that the posture-related component becomes less prominent over time [9] and chronic disease involves a significant degree of central sensitization and stigmatization. This could be part of the explanation behind the difference between this and other series [7–10]. Worth mentioning is that Ferrante et al. and Berroir et al. only treated patients with purely orthostatic headache (40/42 and 30/30), and Chung et al. treated patients markedly younger and with a shorter delay than the patients in this study. Our patients had more mixed symptoms probably again reflecting a longer diagnostic delay contributing to the differences in results.

This study contains some apparent weaknesses. It is unblinded, retrospective and contains relatively few patients. The ever present lack of control for spontaneous recovery also applies to this study, but since the Danish Headache Center is a tertiary center, all the patients have received conservative treatment elsewhere, including a watchful wait. Patient compliance in returning post-treatment headache diaries proved to be a considerable challenge. Also the level of detail (or lack of) lead to contact to the treated patients to clarify headache diary entries.

The headache diary, in turn, can also be considered a strength in addition to a systematic prospective approach since it allows us to monitor patients more precisely on several parameters. In turn this allows us to define more effect parameters than seen in other studies. Furthermore, as this is a “before-and-after” study, it contributes to considerable clinical and statistical validity.

As conclusion, in this pilot study of very long lasting low CSF pressure headache we found a significant reduction in AUC, a clinically significant reduction in intensity but also a significant increase in days with use of medication after being treated with EBP. Additionally, we recorded a trend towards a reduction of headache duration but the very long lasting diagnostic delay may have compromised the outcome. Increased awareness and early diagnosis of low CSF pressure headache is hereby emphasized and a controlled larger randomized study is needed to confirm the results. However, the present results allow us to conclude that EBP in treatment-refractory low CSF pressure headache can be considered as a treatment option.
